# Physiochemical analyses and molecular characterization of heavy metal-resistant bacteria from Ilesha gold mining sites in Nigeria

**DOI:** 10.1186/s43141-023-00607-5

**Published:** 2023-12-22

**Authors:** Glory Jesutomisin Ojo, Olugbenga Samson Onile, Abdul Onoruoiza Momoh, Bolaji Fatai Oyeyemi, Victor Omoboyede, Adeyinka Ignatius Fadahunsi, Tolulope Onile

**Affiliations:** 1 Department of Biological Sciences, Biotechnology Programme, Elizade University, P.M.B, 002 Ilara-Mokin, Ilara-Mokin, 340271 Nigeria; 2 Department of Biological Sciences, Microbiology Programme, Elizade University, Ilara Mokin, P.M.B, 002, Ilara-Mokin, 340271 Nigeria; 3 Department of Science Laboratory Technology, Molecular Biology Group, The Federal Polytechnic, Ado-Ekiti, Ekiti, Nigeria; 4 Department of Biochemistry, School of Life Sciences (SLS), Federal University of Technology Akure, P.M.B 704, Akure, Nigeria

**Keywords:** Heavy metals, Heavy metal-resistant bacteria, Gold mining, Molecular characterization;, Tolerance

## Abstract

**Background:**

The contribution of the processes involved and waste generated during gold mining to the increment of heavy metals concentration in the environment has been well established. While certain heavy metals are required for the normal functioning of an organism, certain heavy metals have been identified for their deleterious effects on the ecosystem and non-physiological roles in organisms. Hence, efforts aimed at reducing their concentration level are crucial. To this end, soil and water samples were collected from Ilesha gold mining, Osun State, Nigeria, and they were subjected to various analyses aimed at evaluating their various physicochemical parameters, heavy metal concentration, heavy metal-resistant bacteria isolation, and other analyses which culminated in the molecular characterization of heavy metal-resistant bacteria.

**Results:**

Notably, the results obtained from this study revealed that the concentration of heavy metal in the water samples around the mining site was in the order Co > Zn > Cd > Pb > Hg while that of the soil samples was in the order Co > Cd > Pb > Hg > Zn. A minimum inhibitory concentration test performed on the bacteria isolates from the samples revealed some of the isolates could resist as high as 800 ppm of Co, Cd, and Zn, 400 ppm, and 100 ppm of Pb and Hg respectively. Molecular characterization of the isolates revealed them as *Priestia aryabhattai* and *Enterobacter cloacae*.

**Conclusion:**

Further analysis revealed the presence of heavy metal-resistant genes (HMRGs) including *merA, cnrA,* and *pocC* in the isolated *Enterobacter cloacae.* Ultimately, the bacteria identified in this study are good candidates for bioremediation and merit further investigation in efforts to bioremediate heavy metals in gold mining sites.

**Supplementary Information:**

The online version contains supplementary material available at 10.1186/s43141-023-00607-5.

## Background

Various anthropogenic activities, especially mining, have been well reported to negatively affect the environment, these effects are diverse and include the destruction of ecosystems by polluting the aquatic and terrestrial environment and altering soil properties [1]. Apart from the destruction of the physical habitat, mining also causes the loss of biodiversity [2]. Therefore, mining sites cause various toxicological challenges for the surrounding ecosystems and human health. Of all the numerous mining activities ongoing in the world, gold mining activities occur at a high rate due to its economic value. Three basic steps (mining, mineral processing, and metallurgical extraction) are involved in the gold mining process, and over 99% of the ore extracted during gold mining is released into the surrounding environment as waste [3]. Although heavy metals (HM) are elements that occur naturally in the environment, they are however part of the wastes that have been implicated around gold mining sites [4].

Generally, HMs are referred to as elements with atomic weight and a density greater than that of water [5]. They are classified into essential and non-essential based on their functions and importance to biological systems. Notably, essential HM including Copper (Cu), Zinc (Zn), Nickel (Ni), Manganese (Mn), and Iron (Fe) are utilized in various physiological and biochemical functions that are pertinent to the existence and normal functioning of living organisms. Exemplifying this is their role in facilitating enzymatic reactions by serving as enzyme cofactors and their role in the regulation of osmotic balance [6]. Conversely, HMs such as Lead (Pb), Arsenic (As), Cadmium (Cd), Chromium (Cr), and Mercury (Hg) do not perform any physiological role in living organisms; hence, they are referred to as non-essential HMs [7]. Noteworthy, both essential and non-essential HMs are known to pose deleterious effects on plants, animals, microorganisms, and the environment, an effect that has been widely reported to be heavily dependent on the dose and duration [8]. The accumulation of HMs in plants gives rise to numerous adverse effects such as stunted plant growth, reduction in the ability to photosynthesize and undergo mitosis, decrease in enzymatic activity and nutrient intake, as well as chlorosis [9, 10]. The consumption of these plants by humans, in turn, causes negative effects such as immunosuppressive functions, blindness, and neurological damage, as well as playing roles in the etiology of pathologies such as cancer and hypertension [11, 12]. Similarly, the presence of HM in aquatic environments also causes oxidative damage to animals in such environments [13]. HMs also affect the soil and its biota by leading to the loss of soil microbial diversity, change in soil PH and porosity, and reduced microbial enzymatic activities [14].

While exposure to HM results in the loss of soil microbial diversity and reduced enzymatic activities, certain microorganisms particularly bacteria, have developed resistance mechanisms against them, to tolerate the toxic effects they pose. These resistance mechanisms include enzymatic detoxification, exclusion by permeability barrier, intracellular and extracellular sequestration, efflux pumps, active transport, as well as reduction of heavy metal ions and cellular targets [15]. Of note, these resistance mechanisms are often conferred by natural selection or exposure to substrates containing heavy metal ions [16]. *Salmonella*, *Escherichia coli*, and *Rhodobacter sphaeroides* are among the bacteria that have been reported to possess multiple heavy metal resistance genes (HMRGs); hence, they are regarded as suitable for environmental remediation [6, 17].

Profiling of the microorganisms in mining sites could help identify the microorganisms that could be utilized in reducing the concentration of HM in such environments, hence, contributing to the global efforts to reduce pollution caused by HM. Consequently, this study aims to identify microorganisms that are present in the Ilesha gold mining site in Nigeria.

## Methods

### Study area

This study was carried out on a gold mining site located in Ilesa, Osun State, Nigeria. Ilesa is one of the major cities in Osun State having a geographical coordinate of 7.6103° N (Latitude) and 4.7096° E (Longitude). It has six local governments (Ilesa East, Ilesa West, Obokun, Oriade, Atakunmosa East, and Atakunmosa West) with a total estimated population of 620,109, according to the 2006 population census [18]. Ilesha is commonly known to have large commercializable deposits of gold and numerous illegal gold mining sites. It is also known to produce crops such as cocoa, kola nuts, pumpkins, oil palm, and cotton.

### Sample collection

**Fig. 1:**
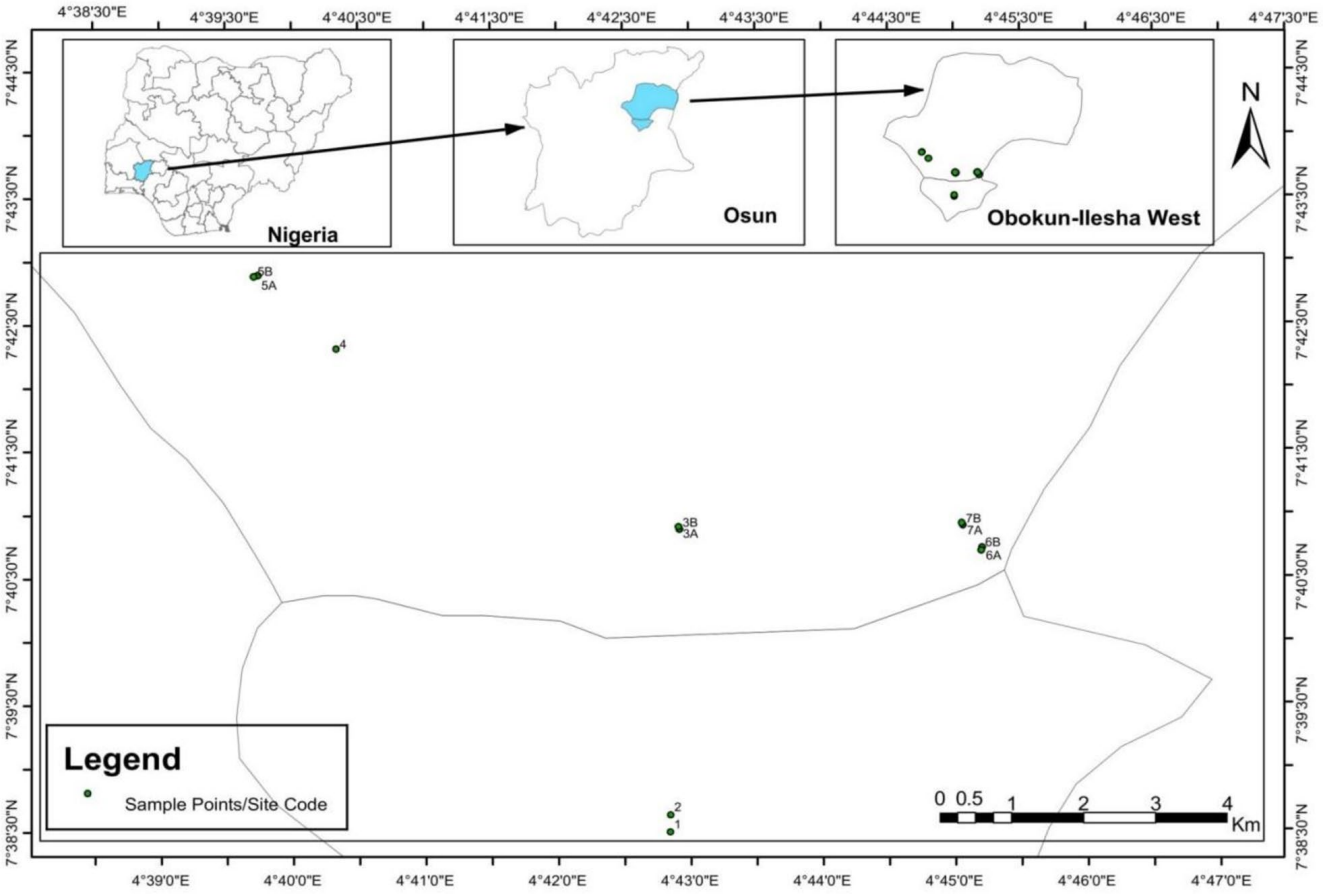
Map of the study area in Ilesa, Osun State, Nigeria

Soil and water samples were collected separately from seven different gold mines located in Ilesa (Fig. 1). The samples were labeled 1A, 1B, 2A, 2B, 3A, 3B, 4A, 4B, 5A, 5B, 6A, 6B 7A, and 7B based on the sampling locations. Table 1 presents the sampling locations and coordinates.

**Table 1:** Sample locations and coordinates

Sample/site code	Locations	Latitude	Longitude	Altitude (m)
1	Isale general, Muroko, Ilesha	N 7°38′29.20416′′	E 4°42′50.50044′′	359
2	Isale general, Muroko, Ilesha	N 7°38′37.38012′′	E 4°42′50.70348′′	355
3A	Iregun ijesha, Osogbo-Ilesha Road	N 7°40′52.56624′′	E 4°42′55.00476′′	364
3B	Iregun ijesha, Osogbo-Ilesha Road	N 7°40′53.85036′′	E 4°42′54.82368′′	342
4	Oora River (left), Oshogbo-Ilesha Road	N 7°42′18.5436′′	E 4°40′19.89228′′	338
5A	Oora River (right), Oshogbo Road	N 7°42′53.38008′′	E 4°39′44.42004′′	322
5B	Oora River (right), Oshogbo Road	N 7°42′52.92972′′	E 4°39′42.73632′′	335
6A	Opo River, Ijaregbe, Ibala Road	N 7°40′43.5612′′	E 4°45′12.2778′′	383
6B	Opo River, Ijaregbe, Ibala Road	N 7°40′42.23136′′	E 4°45′11.95812′′	383
7A	Alatise, Ilesha, Obokun local Government	N 7°40′54.43104′′	E 4°45′3.62196′′	381
7B	Alatise, Ilesha, Obokun local Government	N 7°40′55.26516′′	E 4°45′3.20616′′	381

### Physicochemical analyses of water and soil samples

Various physicochemical properties of the water samples including pH, chloride, conductivity, total hardness, biochemical oxygen demand (BOD), turbidity, phosphate, sulfate, nitrate, bicarbonate, calcium, potassium, magnesium, and sodium were analyzed [19, 20]. Similarly, the pH, conductivity, organic matter, carbon, nitrogen, phosphorus, calcium, magnesium, potassium, and sodium content of the soil samples were also determined using standard methods [21–23].

### Heavy metal analysis

#### Soil digestion

For Zn, Pb, Cd, and Co: the soil samples were pulverized and oven-dried at 500 °C. One gram of the resulting sample was weighed into a 100-mL conical flask and distilled water was added. Subsequently, 10 ml of aqua regia HNO_3_: HCl (3:1) was added to the mixture and boiled with steady heat to almost dryness. The resulting sample was then allowed to cool and filtered, the filtrate was made up to 100 ml with distilled water and subjected to HM analysis.

For Hg: 0.5 g of each soil sample was digested with 10 mL of concentrated HNO_3_ until a clear solution was obtained. The digest was filtered in a 50-mL tube and made up to the 50 mL mark with distilled water, after which it was subjected to HM analysis.

#### Water digestion

For Zn, Pb, Cd, and Co: 100 mL of water sample was measured into a conical flask, and 2 mL of concentrated HNO_3_ and 5 mL of concentrated HCl were added. The resulting mixture was subjected to heating at 900–950 °C till the volume was reduced to 15–20 mL. Ultimately, the volume of the heated mixture was made up to 100 mL and was subjected to heavy metal analysis.

For Hg: 50 mL of the water samples were digested with 5 mL of HNO_3_ until the volume was reduced to 20 mL and the volume was made up to 50 mL with distilled water. It was then subjected to heavy metal analysis.

#### Determination of heavy metals in samples using atomic absorption spectrophotometer (AAS)

An atomic absorption spectrometer (AAS) was utilized to determine the heavy metal content of the samples in accordance with APHA 20th Edition 3111B and 3111D, ASTM D3561, and ASTM D5198. Direct aspiration of the digested liquid sample in an acidic medium into an air/acetylene or nitrous oxide/acetylene flame at specified wavelengths for each of the heavy metals under investigation was carried out to determine the concentrations of heavy metals in the samples [24].

### Enumeration of bacterial loads in soil and water samples

One gram of soil and water sample was measured into 100 mL of distilled water each and 1 ml was transferred into another test tube containing 9 mL of sterile distilled after thorough shaking. Ultimately, making a serial dilution of up to 10^−4^ [25]. One milliliter of the sample from dilution 10^−3^ was utilized for the pour plate technique to enumerate the microbial load of the samples while the streaking method was used to isolate bacteria colonies of pure culture [26].

The bacterial colony count was determined by multiplying the number of counts with the dilution used and expressed as colony-forming units per milliliter (cfu/mL) of water and colony-forming units per gram (cfu/g) of soil [27].

### Characterization of bacterial isolates

The morphological characteristics of the bacteria colonies such as edges, shape, and surface were observed and recorded [28]. The identification and characterization of bacteria isolates were performed by carrying out grams staining and other relevant biochemical tests which include catalase test, coagulase test, indole production test, citrate utilization test, and sugar fermentation [29, 30].

### Minimum inhibitory concentration (MIC) test

The minimum inhibitory concentration (MIC) tests of heavy metals were performed using the broth macrodilution method of the Clinical and Laboratory Standards Institute [31]. The individual isolate was cultured in nutrient broth for 18–20 h at 37 °C. The bacterial suspension was diluted to a 0.5 McFarland standard with sterile saline water and inoculated into a media containing different concentrations of the heavy metal salts. Different Part per million (PPM) concentrations of the following heavy metal salts were prepared for the test: mercury chloride (HgCl_2_), cobalt chloride (CoCl_2_), cadmium chloride (CdCl_2_), zinc sulfate (ZnSo_4_), and lead acetate [Pb(C_2_H_3_O_2_)_2_]. Concentrations of 12.5, 25, 50, 100, 200, 400, 600, and 800 ppm were used for cadmium, cobalt, zinc and lead respectively [16], while concentrations of 6.25, 12.5, 25, 50, 100 and 200 ppm were used for mercury. After inoculating each isolate to media containing different concentrations of heavy metal salt, the mixtures were incubated at 35 °C for 18–20 h and MIC was recorded as the lowest concentration that visibly inhibits bacterial growth [16]. Positive and negative controls were also prepared for this test. The positive control consisted of the medium and the bacteria isolate, while the negative control consisted of the medium and the heavy metal salt only [32].

### DNA extraction and PCR amplification of HMRGs

DNA was extracted from the bacteria isolates of the bacteria that were able to grow in high concentrations of heavy metals using the ZymoBIOMICS™ DNA Miniprep kit [16]. The genotyping for the heavy metal resistance gene in isolated bacteria was performed by PCR using gene-specific forward and reverse primers having similar annealing temperatures of 57 °C (Table 2). The methods and PCR primers (Table 3) used were selected from a previously published study [16]. The PCR products were subjected to gel electrophoresis using 1.5% agarose gels and were viewed on a gel documentation system.

**Table 2:** Primers used for the amplification of HMRGs

Heavy metal	Genes	Primer sequences	Size	Annealing temperature (^0^C)
Cd	*cadD*	AATTGCAAGTTGTGGTGCAG CCCACACCAGGAATTCTAGC	155	57
Co, Ni	*cnrA*	CCTACGATCTCGCAGGTGAC GCAGTGTCACGGAAACAACC	422	57
Cu	*pcoC*	TTCTTACAGGTGGCCTCGTT CCGGTAATAGGGTGCGTATC	333	57
Pb	*pbrT*	AGCGCGCCCAGGAGCGCAGCGTCTT GGC TCG AAG CCG TCG AGR TA	448	57
Hg	*merA*	GAGATCTAAAGCACGCTAAGGC GGAATCTTGACTGTGATCGGG	1011	57

**Table 3:** 16S rRNA primer sequences

Name of primer	Target	Sequence (5′ to 3′)
16S-27F	16S rDNA sequence	AGAGTTTGATCMTGGCTCAG
16S-1492R	16S rDNA sequence	CGGTTACCTTGTTACGACTT

### Molecular identification of bacteria isolates and phylogenetic analysis

Genomic DNA was extracted from the cultures using the Quick-DNA™ Fungal/Bacterial Miniprep kit (Zymo research catalog number D6005). The 16S rRNA target region was amplified using OneTaq Quick-load 2X Master Mix 9NEB, (Catalogue number M0486) with primers presented in Table 3. The PCR products were run on a gel and cleaned up enzymatically using the EXOSAP method. The purified fragments were sequenced in the forward and reverse direction using the ABI 3500XL Genetic Analyzer (Applied Biosystems, ThermoFisher Scientific). BioEdit Sequence Alignment Editor Version 7.2.5 was used to analyze the ab1 files generated by the ABI 3500XL Genetic Analyzer and the results obtained were analyzed by BLASTn search. Subsequently, Molecular Evolutionary Genetics Analysis (MEGA) 11 software was employed to perform phylogenetic analysis using the Test Maximum Likelihood method and the bootstrap consensus tree was inferred from 100 replicates [33].

### Statistical analysis

The statistical analysis of results obtained was done using Statistical Package for Social Sciences (SPSS), version 25 using analysis of variance (ANOVA) on Windows 10 at a confidence level of 95%.

## Results

### Physicochemical properties of water samples

The results of the analysis of various physicochemical parameters carried out on the water samples are presented in Table 4. Notably, the pH values of the water samples ranged from 6.2 to 7.2 with sample 1 having the highest pH value of 7.2 while sample 2 had the lowest pH value of 6.2 (Table 4). Analysis of the conductivity of the water samples revealed the conductivity values ranged from 84.4 µS/cm to 540 µS/cm, with Samples 6B and 5A having the lowest and highest values respectively (Table 4). The result of the analysis of the biochemical oxygen demand (BOD) of the water samples revealed the values ranged from 50.0 Mg/L to 108.5 Mg/L, with samples 5A and 5B having the lowest and the highest values respectively. The turbidity values of samples 1, 3B, 4A, 4B, 5B, 6B, and 7A were the lowest with a value of 10.0 Nephelometric turbidity unit (NTU), while sample 5A was the highest with a value of 28.5 NTU. The results of the total hardness of the water samples ranged from 0.32 to 1.00 mg/L, the lowest and the highest values recorded were from samples 6A and samples 4A respectively (Table 4).

**Table 4:** Physicochemical properties of the water samples

Physicochemical parameters			Samples										
	1	2	3A	3B	4A	4B	5A	5B	6A	6B	7A	7B	WHO
pH	7.2	6.2	6.4	6.5	7.1	6.8	6.7	7.1	6.4	6.6	6.5	6.6	6.5–8.5
Conductivity µS/cm	196.2	88.5	277	253	252	166.5	540	175.5	87.6	84.4	158.5	128.3	750
BOD (Mg/L)	60.0	91.0	52.0	53.5	60.0	60.5	108.5	50.0	55.0	60.5	60.0	62.0	5.00
Turbidity (NTU)	10.0	24.5	11.5	10.0	10.0	10.0	28.5	10.0	10.5	10.0	10.0	10.0	5
PO_4_^3−^(Mg/L)	0.3	1.4	0.2	0.2	0.2	0.2	2.31	0.2	0.3	0.2	0.2	0.2	5.0
SO_4_^2−^(Mg/L)	150	110	130	115	180	180	240	140	120	140	140	110	250
NO_3_^−^(Mg/L)	56	89	50	47	25	30	22	30	28	27	28	49	50
HCO_3_^−^(Mg/L)	355	300	331	320	408	411	590	320	302	353	346	303	125–130
Cl^−^(Mg/L)	2.6	3.4	2.0	2.4	2.8	2.9	3.4	1.6	2.2	2.4	2.3	2.0	250
Na^+^ (Mg/L)	47.8	50.0	20.6	20.0	29.5	28.0	10.5	28.6	28.0	26.4	27.0	25.0	200
K^+^ (Mg/L)	4	20	6	5	8	8	22	7	6	8	7	7	12
Ca^2+^ (Mg/L)	73	94	62	51	59	70	212	60	70	64	68	62	75
Mg^2+^ (Mg/L)	64	90	60	46	63	69	118	49	54	50	58	44	125
Total hardness	54	69	93	87	100	49	132	50	32	65	42	47	200

Key: *WHO* World Health Organization, 2011, 2022

#### Anion (Mg/L)

Further analysis of the water samples for the presence of phosphate (PO_4_^3−^) revealed samples 3A, 3B, 4A, 4B, 5B, 6B, and 7A had the lowest phosphate concentration with all having the same value of 0.2 Mg/L, while sample 5A had highest concentration with a value of 2.31 Mg/L. The concentration of sulfate (SO_4_^2−^) in the water samples ranged from 110 Mg/L to 240 Mg/L, samples 2 and 7B had the lowest concentration while sample 5A had the highest concentration (Table 4). The lowest nitrate (NO_3_^−^) concentration was observed in sample 5A while sample 2 had the highest values of 22 Mg/L and 89 Mg/L respectively. The highest value observed for bicarbonate (HCO_3_^−^) in water samples was that of sample 5A with 590 Mg/L while the lowest concentration was for sample 2 with a value of 300 Mg/L (Table 4). The concentration of chloride ion (Cl^−^) in water samples ranged from 1.6 to 3.4 Mg/L with sample 5B having the lowest concentration of chloride ion while samples 2 and 5A had the highest chloride concentration.

#### Cations (Mg/L)

The concentration of sodium ion (Na^+^) in water samples ranged from 10.5 to 50.0 Mg/L, with sample 2 having the highest Na^+^ concentration of 50.0 Mg/L while sample 5A had the lowest Na^+^ concentration of 10.5 Mg/L. The highest potassium ion (K^+^) concentration was observed in sample 5A with a value of 22 Mg/L while the lowest concentration was observed in sample 1 with a value of 4 Mg/L. Further analysis also revealed sample 5A had the highest concentration of calcium ions with a value of 212 Mg/L while sample 3B had the lowest concentration with a value of 51 Mg/L. The highest magnesium (Mg^2+^) concentration of 118 Mg/L was observed in sample 5A and the lowest Mg^2+^ of 46 Mg/L was observed in sample 3B (Table 4).

### Physicochemical properties of soil samples

Table 5 presents the results of the various physicochemical analyses carried out on the soil samples. The pH values of the soil samples ranged from 5.1 to 6.9 with sample 7B having the highest pH value of 6.9, while sample 3A had the lowest pH value of 5.1. The conductivity of the soil samples ranged from 17.8 to 126.1 µS/cm, with samples 6A and samples 7A having the lowest and the highest conductivity values respectively. Sample 6B was observed to possess the highest percentage carbon concentration while sample 5B had the lowest value. The percentage concentration of organic matter ranged from 0.30 to 0.83%, with samples 4A and 7A having the lowest and the highest values respectively (Table 5). The percentage concentration of nitrogen in the soil samples ranged from 0.02 to 0.26%. Notably, samples 4A, 4B, 5A, 5B, and 6A had the lowest values while sample 3A had the highest value. Sample 6B had the highest phosphorus concentration of 6.28 Mg/Kg, while sample 3B had the lowest concentration of phosphorus with 4.0 Mg/Kg.

**Table 5:** Physicochemical properties of soil samples

		Samples												
Physicochemical parameters	1A	1B	2A	2B	3A	3B	4A	4B	5A	5B	6A	6B	7A	7B
pH	6.4	6.1	6.3	5.5	5.1	5.6	5.7	6.5	6.4	6.3	5.8	6.1	6.6	6.9
Conductivity µS/cm	36.7	66.2	37.8	61.1	105.2	40.1	34.6	28.5	60.4	29.8	17.8	30.1	126.1	31.1
Carbon (%)	0.41	0.43	0.38	0.30	0.41	0.42	0.38	0.39	0.42	0.29	0.63	0.65	0.55	0.38
Organic Matter (%)	0.33	0.36	0.44	0.42	0.31	0.34	0.30	0.33	0.38	0.40	0.41	0.40	0.83	0.36
Nitrogen (%)	0.03	0.04	0.04	0.03	0.26	0.03	0.02	0.02	0.02	0.02	0.02	0.03	0.05	0.03
Phosphorus (Mg/Kg)	5.11	5.16	6.0	6.03	4.16	4.0	5.01	5.08	5.54	4.83	6.22	6.28	6.18	5.01
Ca^2+^ (CmolKg^−1^)	1.0	1.12	0.96	1.11	1.01	1.12	0.79	0.83	1.04	1.06	1.11	0. 93	1.00	0.81
Mg^2+^ (CmolKg^−1^)	0.72	0.73	0.61	0.60	0.58	0.61	0.69	0.70	0.62	0.64	0.70	0.68	0.55	0.71

#### Cations (CmolKg−1)

The concentration of Ca^2+^ in the samples ranged from 0.79 to 1.12 CmolKg^−1^, with sample 4A having the lowest concentration while samples 1B and 3B had the highest concentration. The concentration of Mg^2+^ in the soil samples ranged from 0.55 to 0.73 CmolKg^−1^ with samples 7A and Sample 1B having the lowest and the highest values respectively. The highest concentration of K^+^ in soil samples was 0.32 CmolKg^−1^ and was observed in sample 6A, while sample 4B had the lowest concentration of K^+^ with 0.20 CmolKg^−1^. The highest concentration of Na^+^ was observed in sample 1B with a value of 0.23 CmolKg^−1^ while the lowest concentration was observed in samples 6B and 7A with a value of 0.12 CmolKg^−1^.

#### Heavy metal analysis of water samples

The results of the analysis of the heavy metals concentration in the water samples are presented in Table 6. The result revealed that the concentration of Cd in the water samples ranged from 0.006 ppm to 0.072 ppm, with samples 4A and 4B having the lowest and highest values respectively. The highest concentration of cobalt (Co) in water samples was observed in samples 2 and 5B with values of 0.036 ppm, while the lowest concentration was observed in sample 7B with a value of 0.004 ppm. Similarly, sample 7B had the lowest concentration of Pb while sample 2 had the highest concentration with 0.183 ppm. The concentration of zinc in the water samples ranged from 0.119 ppm to 0.275 ppm with sample 4A and sample 2 having the lowest and the highest concentration. The concentration of mercury in water samples ranged from 0.516 ppm to 0.658 ppm with sample 5A and sample 7A having the lowest and the highest concentration respectively.

**Table 6:** Heavy metal concentration in water samples

Samples	1	2	3A	3B	4A	4B	5A	5B	6A	6B	7A	7B	WHO	Mean
Cd (ppm)	0.019	0.045	0.012	0.010	0.006	0.072	0.038	0.050	0.035	0.020	0.014	0.015	0.003	0.028
Co (ppm)	0.012	0.036	0.009	0.006	0.018	0.029	0.021	0.036	0.011	0.010	0.006	0.004	0.008	0.016
Pb (ppm)	0.049	0.183	0.026	0.031	0.012	0.067	0.027	0.033	0.019	0.026	0.012	0.009	0.01	0.041
Zn (ppm)	0.172	0.275	0.158	0.137	0.119	0.209	0.187	0.214	0.187	0.192	0.137	0.155	–	0.178
Hg (ppm)	ND	ND	ND	ND	ND	ND	0.658	0.603	ND	ND	0.516	0.549	0.06	0.582

#### Heavy metal analysis of soil samples

Table 7 presents the result of the heavy metal concentrations in the soil samples. Sample 2B was observed to have the highest Cd concentration of 0.146 ppm and sample 7A had the lowest Cd concentration of 0.016 ppm. The concentration of Co ranged from 0.012 ppm which was observed in samples 3A and 6A, to 0.078 ppm which was observed in sample 5A. Sample 2B was observed to possess the highest Pb concentration of 0.612 ppm while sample 7A was observed to possess the lowest Pb concentration of 0.109 ppm. The concentration of Zn in the soil samples ranged between 0.298 ppm to 1.221 ppm with samples 7B and 2A having the lowest and the highest concentration respectively. The concentration of mercury in soil samples ranged from 0.309 ppm observed in sample 7B to 0.796 ppm observed in sample 5A.

**Table 7:** Heavy metals concentration in the soil sample

Samples	1A	1B	2A	2B	3A	3B	4A	4B	5A	5B	6A	6B	7A	7B	US EPA	Mean
Cd (ppm)	0.086	0.080	0.117	0.146	0.069	0.075	0.051	0.095	0.122	0.095	0.030	0.059	0.016	0.022	0.48	0.076
Co (ppm)	0.035	0.045	0.071	0.096	0.012	0.020	0.017	0.046	0.078	0.061	0.012	0.034	0.013	0.017	50	0.039
Pb (ppm)	0.474	0.505	0.596	0.612	0.325	0.311	0.193	0.224	0.371	0.315	0.193	0.252	0.109	0.117	200	0.328
Zn (ppm)	1.064	0.890	1.221	0.756	0.975	0.985	0.610	0.525	0.815	0.701	0.572	0.615	0.320	0.298	1100	0.739
Hg (ppm)	ND	ND	ND	ND	ND	ND	ND	ND	0.796	0.358	ND	ND	0.510	0.309	1.0	0.493

*US EPA* United States Environmental Protection Agency

### Enumeration of bacterial loads in soil and samples

**Fig. 2:**
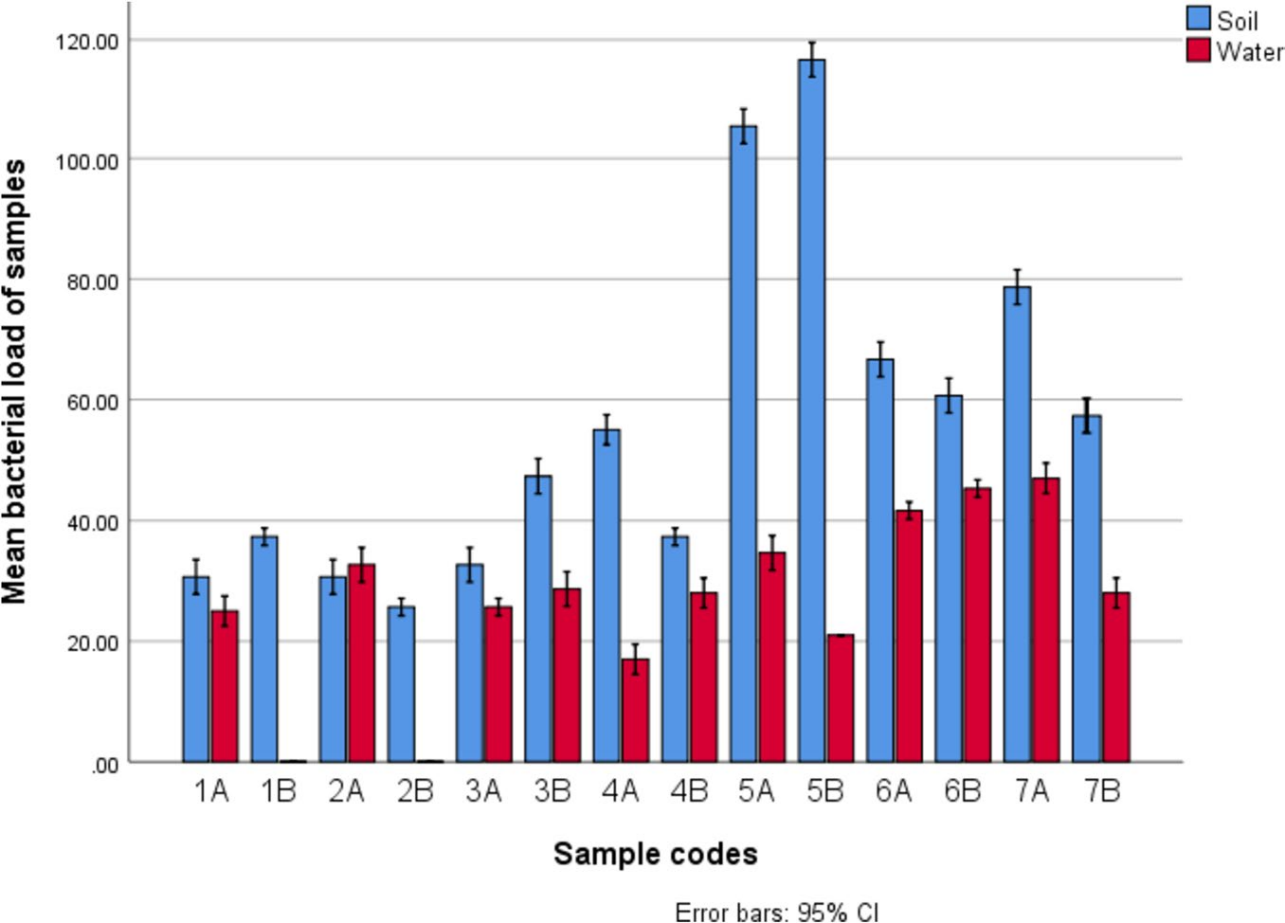
A Comparative Mean bacterial load of Soil and Water samples from the Ilesa Gold mine

The results of the mean bacterial load of each soil and water sample on nutrient agar are presented in Fig. 2. For the soil samples, the results show that sample 5B had the highest bacteria load of 11.67 ± 0.67 × 10^4^ CFU/g, and soil sample 2B had the lowest bacteria load of 2.57 ± 0.33 × 10^4^ CFU/g. Conversely, water sample 7A had the highest bacteria load of 4.70 ± 0.58 × 10^4^ CFU/mL, and the lowest bacteria load of 1.70 ± 0.58 × 10^4^ CFU/mL was observed in sample 4A (Fig. 2).

#### Characterization of bacterial isolates

A total of ten bacteria isolates were purified and isolated from the soil and water samples. Table 8 presents the result of the analysis of the morphological characteristics of the bacteria isolated from the soil and water samples. Notably, isolates 1 and 3 are gram-negative cocci bacteria with creamy white colonies and also negative for spore formation. Isolates 2, 6, 8, and 10 are gram-positive rod bacteria that were also positive for spore formation. Isolate 4 is a gram-positive cocci bacterium, positive for spore formation and creamy white. Isolates 5, 7, and 9 are gram-negative rod bacteria and are also negative for spore formation (Table 8). The probable identity of the 10 bacteria isolates was determined after subjecting the isolates to Gram’s stain reaction and various biochemical tests including coagulase, catalase, indoles, motility, and sugar fermentation (Table 9). The occurrence and distribution of isolates are presented in Supplementary Tables S1 and S2.

**Table 8:** Morphological characteristics of bacteria isolates

Isolate No	Shape	Size	Pigment	Opacity	Elevation	Surface	Edge	Gram’s reaction	Spore formation
1	Irregular	Medium	Creamy-white	Opaque	Flat	Glistening	Wavy	-ve Cocci	-ve
2	Punctiform	Small	White	Opaque	Flat	Glistening	Even	+ ve Rod	+ ve
3	Filamentous	Large	Creamy-white	Opaque	Umbonate	Rough	Filamentous	-ve Cocci	-ve
4	Rhizoid	Medium	Creamy-white	Translucent	Flat	Glistening	Wavy	+ ve Cocci	+ ve
5	Filamentous	Medium	White	Opaque	Convex	Wrinkle	Lobate	-ve Rod	-ve
6	Circular	Large	Yellow	Translucent	Flat	Glistening	Even	+ ve Rod	+ ve
7	Rhizoid	Small	Creamy-white	Transparent	Flat	Rough	Filamentous	-ve Rod	-ve
8	Irregular	Medium	White	Translucent	Flat	Glistening	Wavy	+ ve Rod	+ ve
9	Filamentous	Medium	White	Opaque	Convex	Wrinkle	Lobate	-ve Rod	-ve
10	Filamentous	Medium	Creamy-white	Opaque	Flat	Dull, rough	Filamentous	+ ve Rod	+ ve

Key: + ve (positive); -ve (negative)

**Table 9:** Biochemical and sugar fermentation results of isolates

Isolate no	Simon citrate	Catalase	Coagulase	Indole	Fructose	Glucose	Lactose	Maltose	Galactose	Sucrose	Xylose	Mannitol	Inositol	Arabinose	Probable identity
1	+	+	−	+	+	+	−	+	−	−	−	−	−	+	*Chromobacterium violaceum*
2	−	−	+	+	+	+	−	+	+	+	−	+	−	+	*Thiobacillus*
3	−	−	+	+	+	+	−	+	+ +	−	−	+	−	+	*Klebsiella*
4	−	−	−	+	+	+	−	+	−	+	−	+	−	+	*Rhodopirellula*
5	−	+	+	+	+	+	−	+	+	+	−	+	−	+	*Pseudomonas fluorescens*
6	−	+	−	+	+	+	−	+	−	+	−	+	−	+	*Bacillus subtilis*
7	+	−	−	+	+	+	−	+	+	+	−	+	−	+	*Enterobacter*
8	−	+	+	+	+	+	−	+	+	+	−	+	−	+	*Proteus*
9	+	+	+	+	+	+	−	+	+	+	−	+	−	+	*Pseudomonas plecoglossicida*
10	−	+	+	+	+	+	−	+	−	−	−	+	−	+	*Bacillus*

Key: +  = positive; −  = negative, +  +  = fermentation with gas production

### Minimum inhibitory concentration (MIC)

All 10 bacteria isolates were subjected to a minimum inhibitory concentration test to determine the lowest concentration of heavy metals that visibly inhibits the growth of each bacterial isolate. The MIC values of lead, cobalt, cadmium, zinc, and mercury for bacteria isolates are presented in Table 10.

**Table 10:** Minimum inhibitory concentration of heavy metals (ppm)

Isolates	Pb	Co	Cd	Zn	Hg
1	200	600	600	800	25
2	400	800	800	800	100
3	200	600	600	800	25
4	400	400	400	800	50
5	400	800	400	800	100
6	400	800	800	800	100
7	600	800	800	800	100
8	200	800	600	600	50
9	400	800	800	800	100
10	200	600	600	800	50

Key: *Cd* cadmium, *Co* cobalt, *Pb* lead, *Zn* zinc

### PCR amplification of heavy metals resistance genes (HMRGs)

**Fig. 3:**
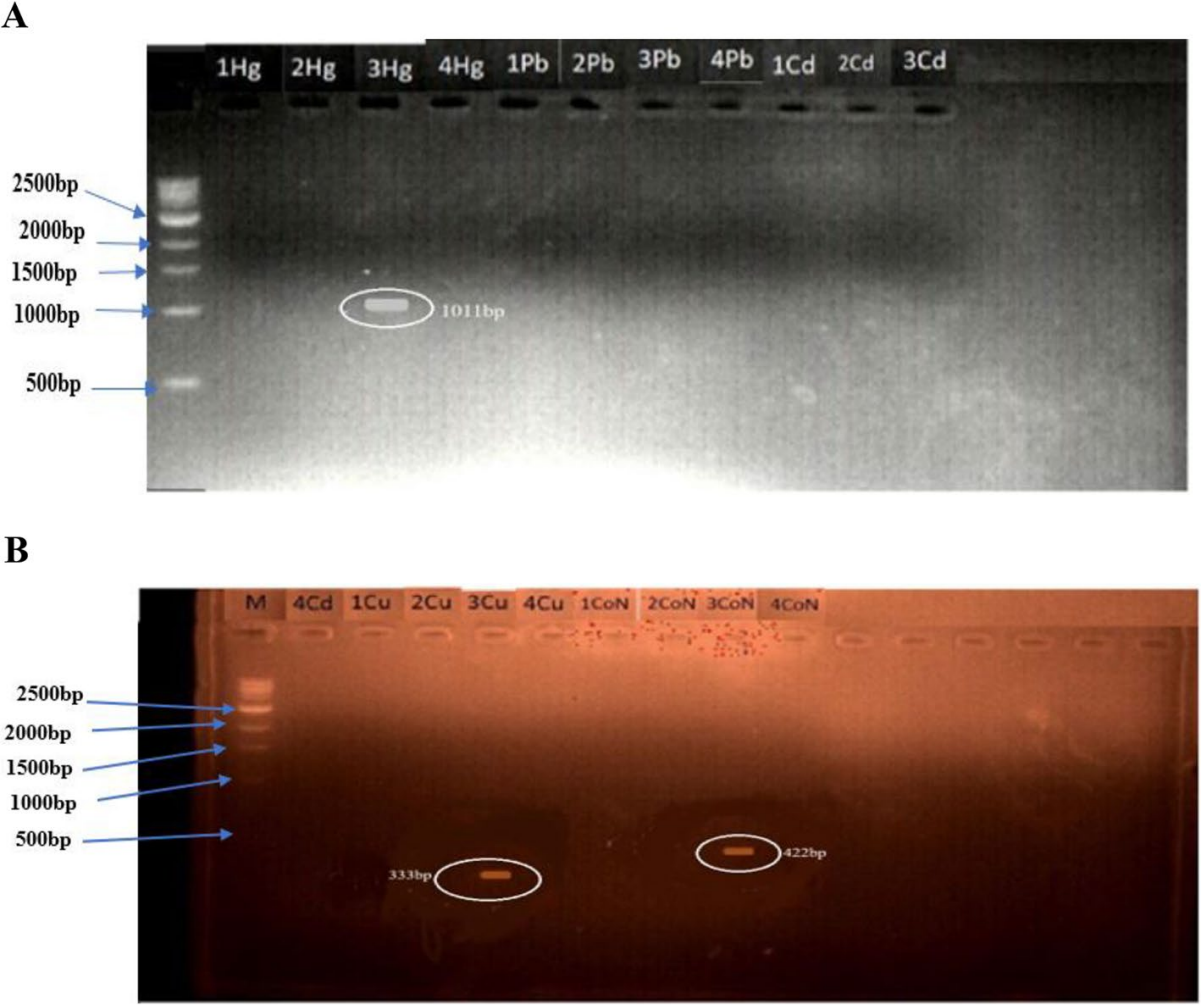
Amplified *HMRGs* in Isolated Bacteria 2, 6, 7, and 9. Key: **A** (amplified mercury *HMRGs* in isolate 7); **B** (amplified cobalt, nickel, and copper *HMRGs* in isolate 7); 1: (Isolate 2) 2: (Isolate 6) 3: (Isolate 7) 4: (Isolate 9); Hg (mercury); Pb (lead); Cd (cadmium); Cu (copper); CoN (cobalt and nickel), M: DNA marker/ladder (1 Kb)

DNA samples were extracted from four bacteria isolates including isolates 2, 6, 7, and 9. DNA was extracted from these four bacteria isolates because they were able to grow at high concentrations of heavy metals that they were subjected to. Isolate 2, 6, 7, and 9 were all genotyped for *HMRGs* with a focus on *merA* (mercury), *pcoC* (copper), *pbrT* (lead), *cadD* (cadmium), and *cnrA* (cobalt and nickel). The *merA, pcoC, pbrT, cadD*, and *cnrA* genes amplified at 1011 bp, 333 bp, 448 bp, 155 bp, and 422 bp respectively. Only isolate 7 amplified for *merA, cnrA*, and *pocC* while all other isolates 2, 6, and 9 did not amplify for any of the *HMRGs* (Fig. 3A, B).

### Molecular identification by 16S rRNA analysis

A total of three isolates (isolates 6, 7, and 9) were subjected to 16S rRNA sequencing, and the identities of the isolates are presented in Table 11. The 16S rRNA sequencing and BLAST result confirmed the identity of isolate 6 to be *Priestia aryabhattai* B8W22 while isolates 7 and 9 are *Enterobacter cloacae subsp. dissolvens* strain LMG 2683 (Table 11).

**Table 11:** BLAST result obtained based on the 16S rRNA sequences of the isolates

Isolates	6	7	9
Predicted organism	*Priestia aryabhattai B8W22*	*Enterobacter cloacae subsp. dissolvens strain* LMG 2683	*Enterobacter cloacae subsp. dissolvens strain LMG 2683*
Query coverage	99.00%	99%	100%
Percentage ID	98.53%	89.17%	96.59%
GenBank accession	NR_115953.1	NR_044978.1	NR_044978.1

**Fig. 4:**
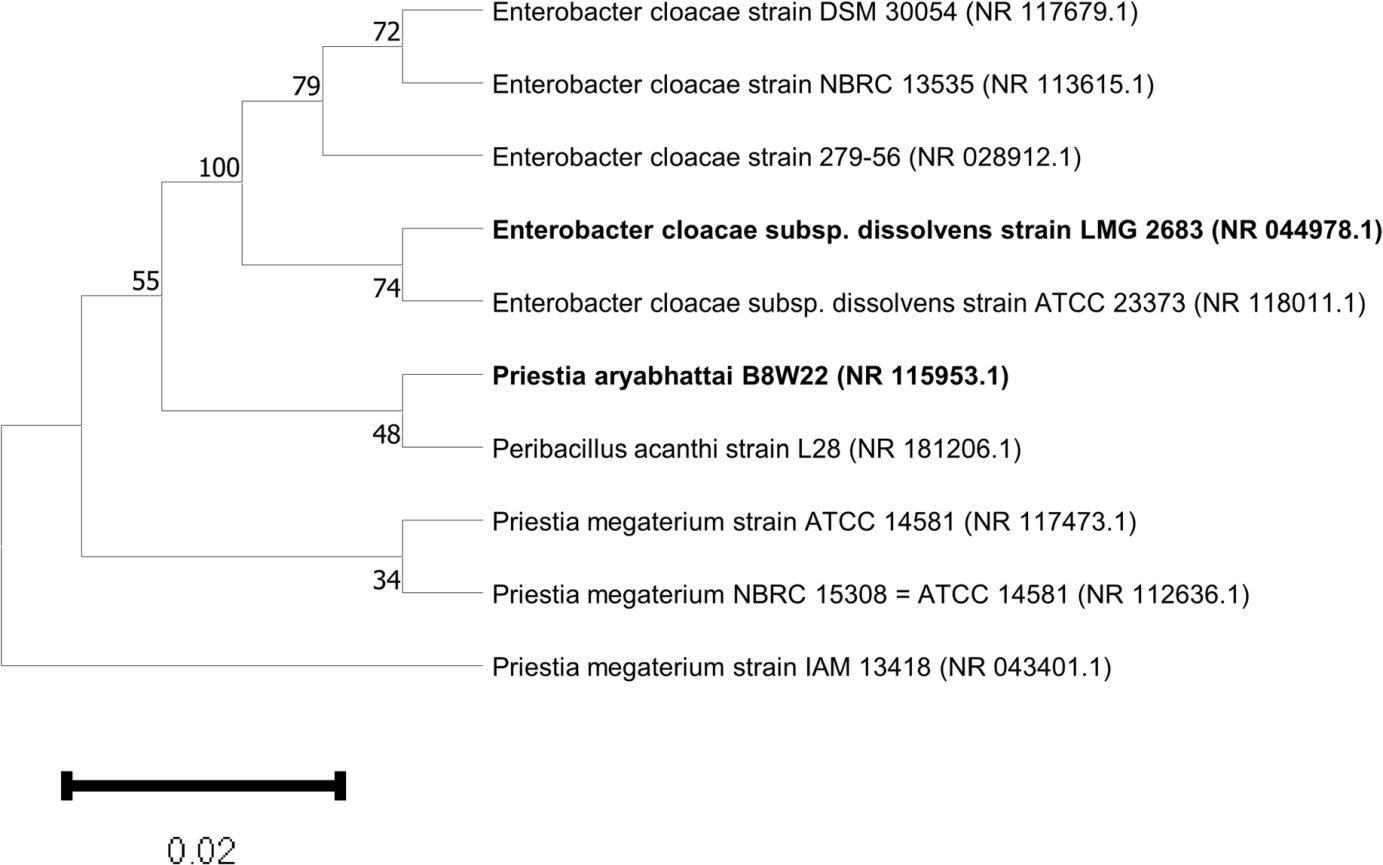
The phylogenetic relationship of *Priestia aryabhattai* B8W22 and *Enterobacter cloacae subsp. dissolvens* strain LMG 2683 constructed using MEGA 11 software

As presented in Table 11 and Fig. 4, isolate 6 was found to bear the closest phylogenetic relationship to *Priestia aryabhattai* B8W22 with a percentage identity of 98.53% in the 99% sequence covered while isolates 7 and 9 were found to bear the closest phylogenetic relationship to *Enterobacter cloacae subsp. dissolvens strain* LMG 2683 with a percentage identity of 89.17% and 96.59% in the 99% and 100% sequence that was covered in their respective query.

## Discussion

HM contamination of water and soil due to various precious metal mining activities is a huge source of concern due to its potential effect on public health. In this study, soil and water samples from seven different gold mining sites in Ilesha were studied for their HM contamination and HM-resistant bacteria. Analysis of the physicochemical properties of the soil and water samples revealed the values of the pH of all the studied sites ranged between 6.2 to 7.2 while that of the soil samples ranged between 5.1 to 6.9. The pH of the soil samples from some of the study areas was observed to be acidic as reported by Akinfesi et al. for African soils [34], however, the ongoing mining activities were noticed to have increased the pH of some of the study areas most notably site 7B as the pH was found to be approaching the neutral level (Table 5). Further increase of the pH to the alkaline range could result in the accumulation of heavy metals on the soil surface [35]. The range of the values of the pH of the water samples from the study area was found to be within the WHO recommended range except that of sample 2 which was found to be more acidic than the other samples (Table 4). The electrical conductivity (EC) of soil and water samples measured in µS/cm gives the total amount of dissolved salts and minerals present in the water or soil sample. The EC values of the water samples were observed to vary greatly with Sample 5A having the highest value, however, they were all observed to be well below the WHO-recommended value (Table 4). This could be attributed to the higher composition of anions in the water samples which were observed to be much higher than the WHO-recommended value, as well as the lower composition of cations which was also well below the recommended value [36]. For the soil samples, the EC values were observed to be much higher compared to the values reported by Ibrahim et al. but much lower compared to the values reported by Edema et al. in mining sites located in different geographical regions in Nigeria [27, 37]. Spatial variation, varying intensities, and levels of gold mining activities could be the probable reason for this as this phenomenon also affects soil properties within the close range [27, 38]. The biochemical oxygen demand (BOD) of all the water samples was found to be well above the WHO-recommended limit (Table 4). Notably, high BOD values indicate high levels of contaminants and result in bacteria requiring more oxygen to degrade the contaminants [39]. The high BOD values of the samples in this study are suspected to be a result of the discharge of heavy metal tailings from the mining sites. These heavy metal tailings contain heavy metals with low- or non-degradability [40]. Assessment of the turbidity profile of the water samples revealed that the values of the turbidity were at least twice that of the WHO recommended value (Table 4). Similar values were reported by Rakotondrabe et al. in a mining site in Cameroon, and it was attributed to soil leaching and deforestation around the mining areas [38]. The highest percentage of carbon and nitrogen were 0.65 and 0.26 respectively, with some samples having as low as 0.02% nitrogen. This depicts that the soil has poor carbon and nitrogen content which is due to the ongoing mining activities which involve the removal of the topsoil and replacement with the soil beneath, a phenomenon which reduces the carbon and nitrogen content of the soil [41]. In tandem with the results of this study is a study by Ibrahim et al. at a gold mining site in Zamfara, Nigeria, in which similar levels of carbon and nitrogen were reported [27].

The concentration of heavy metals in water and soil samples from the mining site under study was also assessed and the results are presented in Tables 6 and 7. The order of abundance of the heavy metals in the water samples based on the mean values of their concentration was: Co > Zn > Cd > Pb > Hg. Conversely, the order of abundance of the heavy metals in the soil samples based on the mean values of their concentration was: Co > Cd > Pb > Hg > Zn. Notably, values of the concentration of some of the water samples from the mining site were found to be higher than the WHO recommendation, with tailings from gold extraction and chemicals used during the gold extraction process suspected to be the probable sources of these heavy metals [42, 43]. Results of the assessment of the heavy metal concentration in the soil samples were much lower compared to values reported in similar studies conducted on heavily metals polluted soils in Nigeria [44, 45]. However, variation in mining activity levels is suspected to be the reason for the wide differences.

Bacterial counts recorded from the soil and water from the mining sites revealed Sample 5B to possess the highest bacteria load of 11.67 × 10^4^ CFU/g for the soil samples while Sample 7A had the highest bacteria load of 4.70 × 10^4^ CFU/ml. As evident from the results, the bacteria counts obtained from the soil and water samples were low and this is likely to be a result of gold mining activity and deposition of tailings in water samples, hence, resulting in the stifling of the microbial community in the sites. However, the presence of resistant species in the sites cannot be overlooked. Ten heavy metal-tolerant isolates were recovered of which preliminary identification analysis revealed five to be Gram-positive while the other five were Gram-negative. Interestingly, studies have shown that Gram-negative bacteria are more tolerant to heavy metals than Gram-positive bacteria, a phenomenon which has been suspected to be due to the ability of their cell wall to interact with the metal ions on the surface and the interface of the bacteria [28, 46]. Further subjection of the bacteria isolates to minimum inhibitory concentration tests revealed isolates 2, 6, 7, and 9 as the isolates capable of tolerating high concentrations of Pb, Co, Cd, Zn, and Hg as evident in Table 9. Precisely, all the bacteria isolates except isolate 7 were not able to grow in the presence of lead at a concentration of 600 ppm. Most bacteria isolate except isolates 2, 6, 7, and 9 had low level of resistance to mercury with no bacteria growth observed in mercury at a concentration of 200 ppm. Corroborating the result of this study is the study of Rahman and Singh in which they reported that the MIC values of Hg for a range of Hg-resistant bacteria were from 50 to 100 mg/L [47]. The ability of Hg to inhibit the growth of these bacteria isolates could be due to its high level of toxicity [48, 49]. Contrastingly, most of the bacteria isolated were resistant to high concentration of Cd and Co, in tandem with this is the result of the study of Terzi and Civelek in which they observed that high concentration of Cd was still being tolerated by their bacteria isolates [17]. HMRGs namely *merA* (mercury), *pcoC* (copper), *pbrT* (lead), *cadD* (cadmium), and *cnrA* (cobalt and nickel) were genotyped in isolates 2, 6, 7, and 9.

Notably, only isolate 7 amplified for *merA, cnrA,* and *pocC* while the other HMRGs were not amplified in any of the other isolates. While the results obtained from the amplification revealed just one isolate as expressing the HMRGs, it is worth noting that the expression of HMRGs is not the only mechanism via which bacteria resist HM. Hence, other mechanisms including the modification of their membrane and metabolic adaptation could be the coping mechanism for the bacteria isolates [50]. Preliminary identification analysis suggested that isolates 2, 6, 7, and 9 are *Thiobacillus, Bacillus subtilis, Enterobacter*, and *Pseudomonas plecoglossicida* respectively. However, further characterization revealed isolate 6 to be *Priestia aryabhattai B8W22* while isolates 7 and 9 were found to be *Enterobacter cloacae subsp. dissolvens strain* LMG 2683. *Priestia aryabhattai*, formerly known as *Bacillus aryabhattai* [51]*,* has been reported to possess heavy metals remediation capacity. In a study by Singh et al., it was reported that the *Priestia aryabhattai* biovolatalized As and they also reported the presence of multiple *ars* genes in the chromosomal DNA of the organism [52]. Interestingly, the *ars*B gene which encodes a transport membrane protein that functions as an efflux pump and extrudes As out of the cell [53], was reported to be present in the organism, *ars*C, which functions in an operon to act as cytoplasmic reductase and reduces As^5+^ to As^3+^ [54], was reported to be present, *arsH* gene, which has been reported to confer a high level of resistance to As (V) and As (III) [55], was also present. Furthermore, the *ars*D gene which encodes a metalloid-responsive transcriptional repressor that is responsible for controlling the expression of *ars* operon [54], *ars*R gene which is responsible for regulating *ars* operon in the presence of As (III) were both present in the organism [56], and *ars*A, an ATPase activated by As (III) were all reported to be present [53]. Also, the organism was reported to upregulate the expression pattern of certain proteins in response to exposure to As, these proteins were found to function in pathways relating to energy metabolism, proline synthesis, and membrane proteins among many others [57]. Hence, it depicts the ability of the bacteria to adjust to As-induced stress conditions. In another study, *Priestia aryabhattai* was also reported to possess the ability to degrade an organophosphate herbicide in a process mediated by the *goxB* gene which encodes a FAD-dependent glyphosate oxidase enzyme [58]. Progressively, this organism is worthy of exploration for bioremediating efforts. Similarly, the heavy metals resistant ability of *Enterobacter cloacae* has been well reported. Exemplifying this is a study by Banerjee et al. in which they reported that *Bacillus megaterium* had very high Pb and Cd removal capacities of 95.25% and 64.17% respectively. Also, Irawati and Tahya reported that *Enterobacter cloacae strains* isolated from the Sukolilo River in Indonesia were able to bioaccumulate copper and had an average biosorption ability of 68% [59].

## Conclusion

Summarily, this study examined the various physicochemical parameters and heavy metal concentrations of soil and water samples from Ilesha gold mining sites. HM-resistant bacteria were also isolated from the samples and were subsequently subjected to a MIC test to ascertain their resistance levels to various heavy metals including Pb, Co, Cd, Zn, and Hg while the molecular characterization of the isolated bacteria was performed. Molecular characterization revealed *Priestia aryabhattai* and *Enterobacter cloacae* as the isolates capable of resisting the high concentration of heavy metals. To the best of our knowledge, this study is the first in which the ability of *Priestia aryabhattai* to resist varying concentrations of Pb, Co, Cd, Zn, and Hg was explored and is also the first to report the presence of heavy metal-resisting *Enterobacter cloacae* in the Ilesha gold mining site. Conclusively, the identified bacteria in this study are worthy of exploration in future attempts aimed at bioremediating heavy metals in gold mining sites and other relevant applications.

### Supplementary Information

**Additional file 1.**

## Data Availability

Not applicable.

## Abbreviations

HMs: Heavy metals
Co: Cobalt
Zn: Zinc
Cd: Cadmium
Pb: Lead
Hg: Mercury
Cu: Copper
Ni: Nickel
Mn: Manganese
Fe: Iron
HMRGs: Heavy metal-resistance gene
MIC: Minimum inhibitory concentration
BOD: Biochemical oxygen demand
AAS: Atomic absorption spectrometer
PCR: Polymerase chain reaction
PO_4_^3^: Phosphate
SO_4_^2^: Sulphate
NO_3_^−^: Nitrate
HCO_3_^−^: Bicarbonate
Cl^−^: Chloride
Na^+^: Sodium
K^+^: Potassium
Mg^2+^: Magnesium
Ca^2+^: Calcium
WHO: World Health Organization
EC: Electrical conductivity
ANOVA: Analysis of variance
SPSS: Statistical Package for Social Sciences
