# Supplementary material for: Physiochemical analyses and molecular characterization of heavy metal-resistant bacteria from Ilesha gold mining sites in Nigeria
Source: J Genet Eng Biotechnol. 2023 Dec 22;21:172. doi: 10.1186/s43141-023-00607-5 (PMC10746654; doi:10.1186/s43141-023-00607-5)
Supplement: Supplementary file 1 — Additional file 1. [file 43141_2023_607_MOESM1_ESM.docx]

| **Bacteria** | 1 | 2 | 3A | 3B | 4A | 4B | 5A | 5B | 6A | 6B | 7A | 7B |
| --- | --- | --- | --- | --- | --- | --- | --- | --- | --- | --- | --- | --- |
| *Pseudomonas fluorescens* | + | - | + | - | - | + | - | + | + | - | + | + |
| *Pseudomonas plecoglossicida* | - | + | - | + | - | + | - | + | - | + | - | + |
| *Klebsiella* | - | + | + | - | + | + | + | - | + | + | + | - |
| *Bacillus subtilis* | + | - | - | + | + | - | - | + | + | + | + | - |
| *Chromobacterium violaceum* | - | - | + | + | + | - | + | - | + | + | - | + |
| *Proteus* | + | + | - | - | + | + | + | + | + | - | - | + |
| *Thiobacillus* | - | + | - | + | - | - | + | - | + | + | - | - |
| *Enterobacter* | + | - | - | + | + | + | - | + | + | + | - | + |
| *Rhodopirellula* | - | - | - | + | + | - | + | + | - | - | + | - |
| *Bacillus* | - | + | - | - | - | + | + | + | - | + | + | + |

**Supplementary Table 1**: Distribution of bacteria isolated from water samples

**Supplementary Table 2**: Distribution of bacteria isolated from soil samples

| **Bacteria** |  | 1A | 1B | 2A | 2B | 3A | 3B | 4A | 4B | 5A | 5B | 6A | 6B | 7A | 7B |
| --- | --- | --- | --- | --- | --- | --- | --- | --- | --- | --- | --- | --- | --- | --- | --- |
| *Pseudomonas fluorescens* |  | - | - | - | + | + | - | - | - | - | + | + | - | + | + |
| *Pseudomonas plecoglossicida* |  | + | - | - | + | - | - | - | + | - | + | - | - | - | + |
| *Klebsiella* |  | + | + | + | + | - | + | + | - | + | - | - | + | + | + |
| *Bacillus subtilis* |  | + | - | + | + | + | + | + | - | - | + | - | - | + | - |
| *Chromobacterium violaceum* |  | - | + | - | - | + | + | + | - | + | - | + | + | - | + |
| *Proteus* |  | - | + | + | - | - | - | + | + | + | - | + | - | - | + |
| *Thiobacillus* |  | + | - | - | + | - | + | - | - | + | - | + | + | - | + |
| *Enterobacter* |  | + | - | + | - | + | - | + | + | - | + | + | + | - | + |
| *Rhodopirellula* |  | + | + | - | - | + | + | - | + | - | + | - | - | + | + |
| *Bacillus* |  | + | - | + | + | - | + | - | - | + | - | + | + | - | + |
